# A multiple technology-based and individually-tailored Sit Less program for people with cardiovascular disease: A randomized controlled trial study protocol

**DOI:** 10.1371/journal.pone.0302582

**Published:** 2024-05-09

**Authors:** Chorong Park, Britta Larsen, Mulubrhan Mogos, James Muchira, Mary Dietrich, Marianna LaNoue, Jason Jean, John Norfleet, Abigail Doyle, Soojung Ahn, Shelagh Mulvaney

**Affiliations:** 1 School of Nursing, Vanderbilt University, Nashville, Tennessee, United States of America; 2 Department of Family Medicine and Public Health, School of Medicine, University of California San Diego, La Jolla, La Jolla, California, United States of America; Baylor College of Medicine, UNITED STATES

## Abstract

Sedentary behavior, a key modifiable risk factor for cardiovascular disease, is prevalent among cardiovascular disease patients. However, few interventions target sedentary behavior in this group. This paper describes the protocol of a parallel two-group randomized controlled trial for a novel multi-technology sedentary behavior reduction intervention for cardiovascular disease patients (registered at Clinicaltrial.gov, NCT05534256). The pilot trial (n = 70) will test a 12-week “Sit Less” program, based on Habit Formation theory. The 35 participants in the intervention group will receive an instructional goal-setting session, a Fitbit for movement prompts, a smart water bottle (HidrateSpark) to promote hydration and encourage restroom breaks, and weekly personalized text messages. A control group of 35 will receive the American Heart Association’s “Answers by Heart” fact sheets. This trial will assess the feasibility and acceptability of implementing the “Sit Less” program with cardiovascular disease patients and the program’s primary efficacy in changing sedentary behavior, measured by the activPAL activity tracker. Secondary outcomes include physical activity levels, cardiometabolic biomarkers, and patient-centered outcomes (i.e. sedentary behavior self-efficacy, habit strength, and fear of movement). This study leverages commonly used mobile and wearable technologies to address sedentary behavior in cardiovascular disease patients, a high-risk group. Its findings on the feasibility, acceptability and primary efficacy of the intervention hold promise for broad dissemination.

## Introduction

Cardiovascular disease (CVD) remains the leading cause of death in the U.S. Half of people who survive a heart attack suffer a second heart attack within three years [1], and 15% die from recurrent heart attacks [2]. Moderate-to-vigorous levels of physical activity (PA) can significantly reduce reinfarction and cardiac mortality [3], and therefore, moderate-to-vigorous levels of PA >150 minutes per week is recommended for CVD patients [4, 5]. However, only 10–21% of CVD patients meet the PA recommendations [6, 7] and they still spend 10–11 hours on sedentary behavior [6, 8–10]. Common barriers to physical activity include fear of reinfarction, shortness of breath, fatigue [11, 12], and time and cost related to exercise [12, 13]. Thus, there is an urgent need to find innovative ways of engaging this population in achieving more active lifestyles.

In people with CVD, sedentary behavior takes up to 70–90% of waking activities [6, 8–10] and 50% of sedentary behavior is prolonged (>30 minutes per bout) [8]. During sedentary time, there is no muscle contraction of the legs, which causes decreasing insulin sensitivity, vascular dysfunction, and activation of low-grade inflammatory cascades [14]. As a result, greater total sedentary time is related to increased CVD risk [15], including decreased high-density lipoprotein (HDL) and increased triglycerides (TG), fasting glucose [16], BMI, and waist circumference (WC) [17]. Prolonged sedentary time is also associated with a 12% higher risk of incidental CVD [18] and an increased risk of CVD mortality regardless of PA levels [19, 20].

Reducing sedentary time with frequent bouts of standing or walking is safe and feasible for patients with chronic diseases [8, 21, 22]. Emerging evidence from meta-analyses and intervention studies conducted in free-living environments indicate significant beneficial relationships of sedentary breaks with BMI, waist circumference, and C-reactive protein (CRP) [23], as well as average glucose levels, insulin and blood pressure (BP) [23–25]. Therefore, frequent sedentary breaks can not only reduce the total sedentary time and increase total standing and walking time, but also break prolonged sitting time, bringing additional cardiometabolic benefits and possibly overcoming common barriers to moderate-to-vigorous PA.

Frequent sedentary breaks can be achieved through an innovative wearable and mHealth approach. For example, utilizing wearables allows people to self-monitor their sedentary time and provides walking prompts to break sedentary behavior. In addition, by utilizing smart water bottle technology, individuals can increase their water intake, which leads them to walk to the restroom more frequently. This can be a natural motivator to break prolonged sitting bouts. A weekly tailored text message based on wearables’ data can motivate participants in reducing sedentary behavior. This multifaceted strategy offers a low-cost, sustainable method for sedentary behavior reduction for CVD patients, who are typically over 60 years old, yet lacks widespread implementation in this age group. While some research has focused on reducing sedentary behavior in CVD patients [8, 9, 26, 27], it predominantly involves individuals already participating in cardiac rehabilitation. Given that these participants tend to be motivated and exhibit higher physical activity and lower sedentary time during rehabilitation, there is a lack of information on how to extend these findings to the broader CVD patient population who may not be as inclined to enroll in cardiac rehabilitation programs.

To respond to these gaps in patient resources and the literature, we developed a multi-technology-based, individually tailored sedentary behavior reduction intervention for people with CVD. The intervention incorporates a coaching session, a wearable fitness tracker (Fitbit Inspire 2), a smart water bottle (HidrateSpark), and individually tailored text messages. The aim of the current paper is to detail the study protocol for the pilot randomized controlled trial (RCT) testing the feasibility, acceptability, and preliminary efficacy of this intervention.

## Materials and methods

### 2.1. Study design

**Fig 1:**
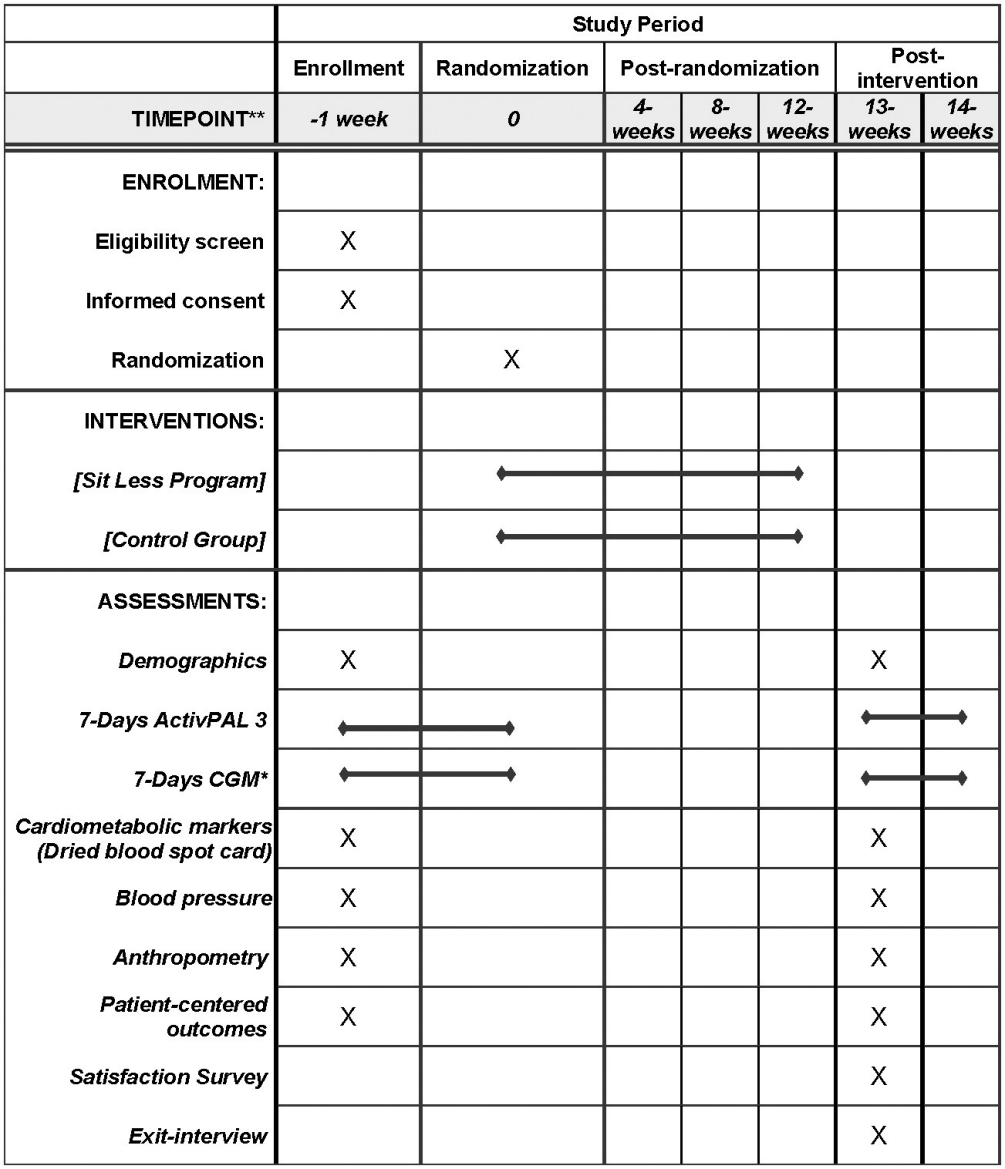
Time schedule of enrollment, interventions, and assessments followed by the SPIRIT 2013 reporting guideline. *CGM = Continuous Glucose Monitoring.

The research study is a single-center, parallel two-group RCT (registered at Clinicaltrial.gov on September 6, 2022, NCT05534256). The research study will adhere to the CONSORT (Consolidated Standards of Reporting Trials) Statement. The study protocol follows the SPIRIT (Standard Protocol Items: Recommendations for Interventional Trials) 2013 Statement (S1 Checklist). The schedule of enrollment, interventions, and assessments is described in Fig 1. This study was reviewed and approved by the Vanderbilt University Medical Center IRB (#220416). Participant recruitment began on September 13, 2022, and is anticipated to end by March 31, 2025.

### 2.2. Participants and sample size

We will recruit 70 patients with CVD who 1) are 18 years or older, 2) have at least one of the following conditions: history of heart attack, coronary/carotid artery disease, ischemic heart disease, peripheral artery disease, a stent placed in the heart or leg or had a coronary artery bypass surgery, 3) self-report of sitting ≥ 8 hours/day, 4) ability to stand and walk, and 5) ownership of a smartphone. We will exclude those who are 1) using an activity tracker, 2) currently participating in exercise or cardiac rehabilitation programs, 3) non-English speaking, 4) classified as unstable (e.g. heart failure, uncontrolled arrhythmia), have kidney disease that limits daily water intake, or any conditions that prevent standing or walking due to physical or cognitive limitations, such as cognitive impairment, severe pain, problems with lower limbs, or a history of surgeries that limit movement, and 5) currently pregnant.

As a pilot/feasibility study, we do not propose a fully powered, hypothesis-testing trial. The primary outcome of this study is the feasibility and acceptability of the Sit Less program, which is not dependent on effect sizes. Therefore, we did not calculate the sample size based on the effect sizes but based on the calculation to identify potential problems in feasibility/acceptability. According to Viechtbauer et al. [28], if the feasibility issue occurs with a 10% chance, a sample size of 30 is required to identify the feasibility issue with a confidence of 95%. The total sample of 70 (35 in the intervention group and 35 in the control group) was estimated to allow sufficient piloting of study protocols and examination of the feasibility of intervention activities and measures to inform future research.

### 2.3. Recruitment

#### 2.3.1. Off-line recruitment

Recruitment flyers and brochures will be posted in cardiology outpatient clinics at Vanderbilt Heart (Nashville, TN, U.S.A) for self-referrals. In addition, the patient care providers will also reach out to their patients and provide their patients with our study brochure to allow the patient to contact the study team. We will also prescreen potential participants using the Epic medical records system for potential patients’ contact information. Initially, the study team will send an opt-out letter to the potential patients. If the patients do not contact the study team within two weeks by email or telephone call to opt-out, the trained research staff will call them, explain the study, and screen for interest and eligibility.

#### 2.3.2. On-line recruitment

We will send a message to potential participants who are registered in the Vanderbilt community-wide email distribution list and ResearchMatch (national web-based recruitment tool). Due to the in-person nature of the protocol, we will only contact those who are living in Nashville or surrounding areas. In the invitation email, there will be a link directing potential participants to the prescreening survey via REDCap. This pre-screening data will be saved into REDCap. Once they meet all the inclusion criteria, the trained research staff will call them to confirm their eligibility.

### 2.4. Baseline visits

**Fig 2:**
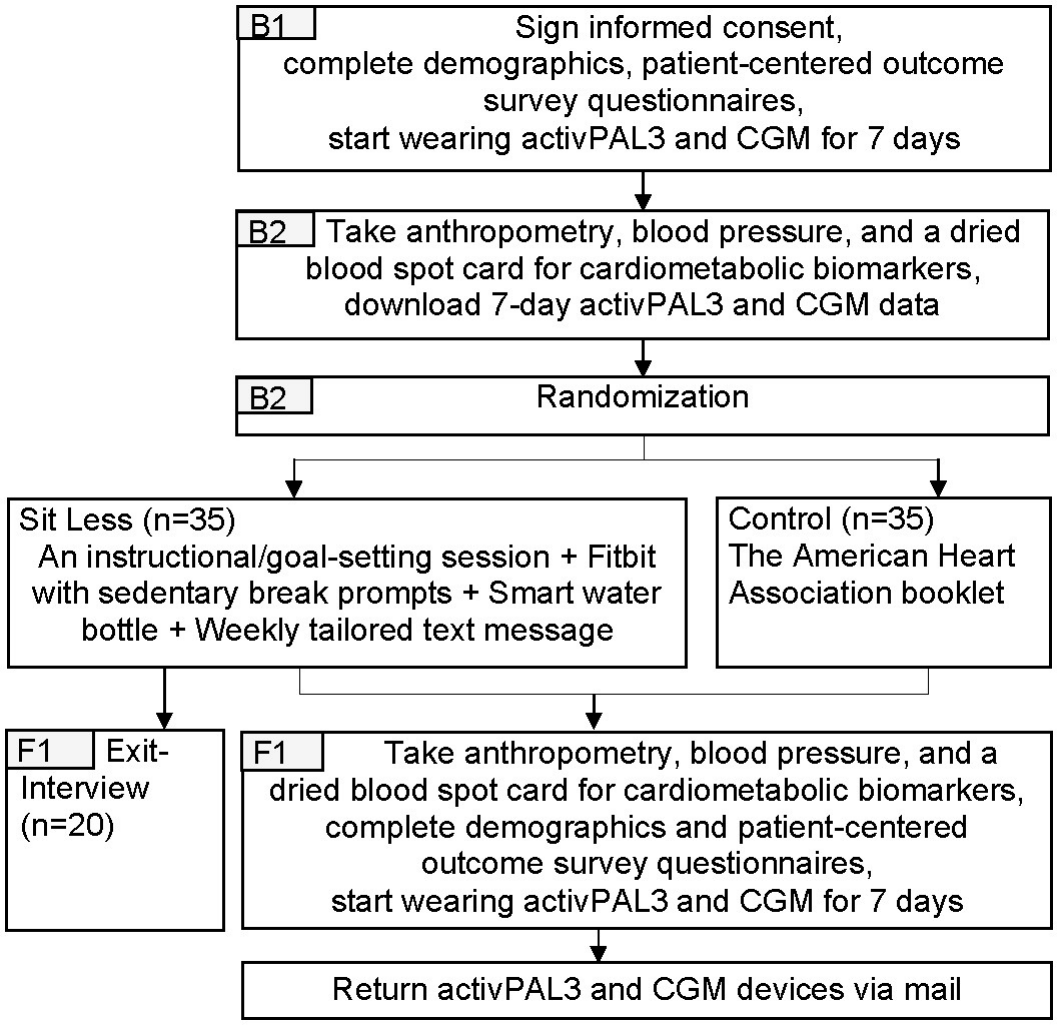
Study flow. B1 = Baseline visit 1, B2 = Baseline visit 2, F1 = Follow-up visit 1, *CGM = continuous glucose monitor.

All interested respondents will be screened by a trained research staff member over the phone to confirm their eligibility, which includes the ability to walk and stand without limitations. Eligible participants will be invited to the initial in-person baseline visit at the Vanderbilt University School of Nursing (VUSN) Research Clinic (Nashville, TN). The study flow is illustrated in Fig 2.

#### 2.4.1. Baseline visit 1

At Baseline Visit 1, eligible individuals will complete the written informed consent with assistance from the trained research staff. Those who are consented and complete the baseline survey questionnaires will commence a 7-day usual physical activity and glucose monitoring period. During this period, all participants will wear an activPAL3 device (PAL Technologies) on the thigh and a continuous glucose monitor (CGM, Abbott FreeStyle Libre Pro) on the upper arm and follow their usual diet and activity. Participants will receive $25 for the completion of the Baseline Visit 1.

#### 2.4.2. Baseline visit 2

After 7 days of activity and glucose monitoring, the participants will return to the VUSN Research Clinic. Baseline anthropometric assessments, blood pressure, and blood collection via finger prick will be completed. The activPAL and CGM will be removed at this visit. Participants will receive $25 for Baseline Visit 2.

### 2.5. Randomization

By using the REDCap randomization module, computer-generated random numbers will be created by a statistician. During Baseline Visit 2, randomization to the intervention and control arms (1:1) will be performed by a study team member. A study team member will click REDCap’s randomization function and each participant’s group assignment will be available online via REDCap. The study team member will immediately inform participants of their group assignment during Baseline Visit 2. Participants and interventionist will not be blinded to the allocation due to the nature of the intervention program. However, outcome assessors and data analysists will be blinded to the allocation.

### 2.6. Intervention

**Fig 3:**
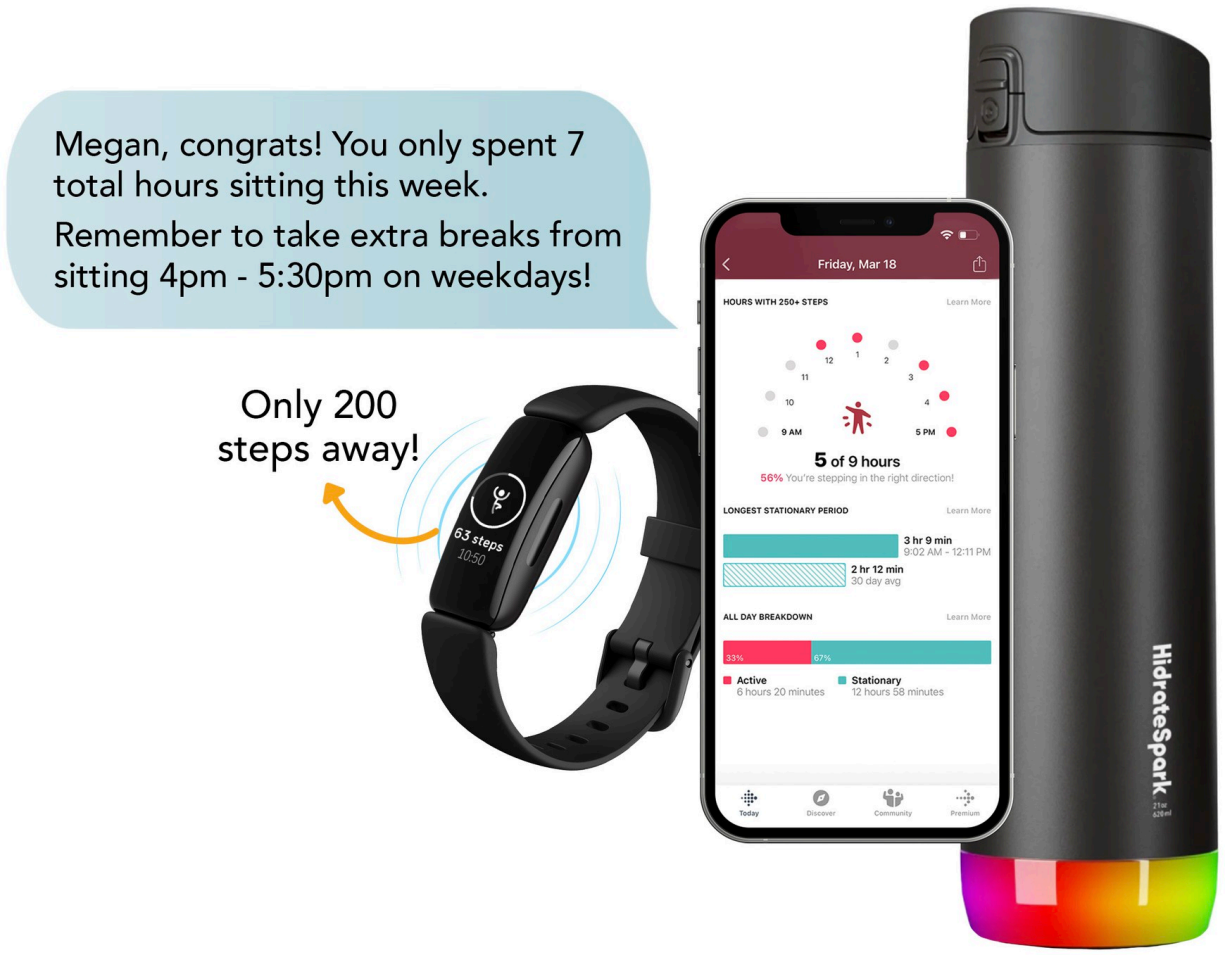
Overview of multiple-technology-based sedentary behavior reduction intervention.

The 12-week intervention (Sit Less program) will consist of four components based on the Habit Formation framework (Fig 3). Previous studies have shown significant (91 minutes/day) reductions in sedentary time following 4 to 24 weeks of intervention [29].

The Habit Formation framework suggests that 12 weeks is required to develop a new habit [30]. We therefore propose a 12-week intervention period.

#### 2.6.1. Theoretical framework

The Sit Less program employs a Habit Formation framework [31] to target the habit-like nature of sedentary behavior, which often occurs without conscious thought or intention [32, 33]. The program focuses on making individuals aware of their sedentary habits and encourages the adoption of new behaviors, such as taking breaks to stand or walk, through consistent practice in specific contexts. According to the Habit Formation framework [31], habit formation involves four phases: motivation, action initiation, behavior repetition in a context, and cue-association building. Through this process, over time individuals may rely on contextual cues rather than conscious self-regulation to initiate a behavior, making it potentially easier to perform and more sustainable with consistent environments [31]. The Habit Formation framework is widely used for sedentary behavior reduction in older adults [32].

The framework guides our intervention by: 1) bringing sedentary behavior into conscious awareness through prompts (e.g., vibrations from a Fitbit), and 2) making a new habit of sedentary breaks (5 minutes of standing/walking every 30 minutes of sitting) through context-dependent repetition (e.g., during TV commercials). To enhance habit formation, behavioral techniques supporting motivation and action planning will be used (Table 1) [34].

**Table 1:** Relationship between intervention components, theoretical constructs, and behavioral techniques.

Intervention component	Theoretical constructs	Behavioral change techniques
**Instructional/ goal setting session**	Motivation, define context and target habitual behavior	Goal setting, instruction on how to perform behavior, information on health consequences, identification of targetable prolonged sitting
**Activity tracker**	Awareness of unconscious sitting habits, gradually develop a new habit of sedentary breaks through repetition, develop a contextual cue-behavior association	Use of sedentary break prompts and cues, context-dependent repetition, self-monitoring, feedback on behavior
**Smart water bottle**	Motivation (I have to get up to go to the restroom), gradually develop a new habit of drinking water	Use of cues through change of bottle color (time to drink water)
**Weekly tailored text message**	Motivation, translate intention into action, enhance contextual cue-behavior association	Feedback on behavior, problem solving, review of goal, setting of new goal

#### 2.6.2. Instructional goal setting session

The PI will provide an instructional goal setting session during Baseline Visit 2. The PI will review the prior 7 days of activPAL3 data and identify targetable prolonged sedentary bouts with the participant. The PI and the participant will set two goals: 1) a sedentary behavior reduction goal aimed at reducing daily sedentary time gradually until they reach the goal of ≥ 120 minutes of sedentary time reduction; and 2) a sedentary break goal of 5 minutes of standing/walking every 1 hour and then increasing its duration to 10 minutes and frequency of the sedentary break to every 30 minutes [35]. These goals have been demonstrated to be feasible in stroke patients and CVD patients [8, 21]. Participants will have the autonomy to set their weekly goals, whether to maintain the same target as the previous week, or reduce sedentary time. We do not recommend reducing sedentary time by more than 30 minutes per week, but adjustments can be made based on the participants’ current sedentary hours and willingness.

In addition, the participant will be given the Ten Top Tips (TTT). The TTT is a booklet developed from the Habit Formation Theory and includes tips to add sedentary breaks into their daily routine [36]. The original TTT booklet [36] was obtained from Dr. Gardner [36], one of the developers of the Habit Formation theory [31], and it was applied to sedentary behavior with permission to revise it for the current study. The original TTT booklet suggested limiting TV watching to no more than one hour at a time and promoting light physical activities such as walking—by leaving the remote control by the TV, standing up during commercials, opting for stairs, and parking at a distance from the entrances of malls or offices [36]. It also included recommendations for stretching and muscle strengthening exercises [36]. We revised the original TTT booklet to include 8 tips, excluding the stretching and muscle training recommendations and incorporating the Fitbit and smart water bottle into daily routines. Although the suggested tips involve only light levels of physical activity, such as standing and walking for 3 to 5 minutes every hour—activities with no more risk than those encountered daily—we will instruct participants to stop these exercises immediately if they feel unwell or experience discomfort, and to promptly seek medical advice.

#### 2.6.3. Activity tracker

A Fitbit Inspire 2 (Fitbit Inc., U.S.A) will be provided to each intervention participant. This device alerts participants with a vibration and message on the watch face when they have been sitting for 60 minutes (i.e., “Move alert”). Participants can select the days and the time ranges to receive the move alerts. Fitbit provides sedentary behavior statistics including the longest sitting period (“Longest Stationary Period”) and total daily sitting hours (“Stationary Hours”) in the app. Participants will be encouraged to check the Fitbit app at the end of the day to monitor their longest sitting period and total daily sitting hours. They will be guided to apply the TTT booklet tips to interrupt their longest sitting period and decrease total daily sitting hours, aiming to achieve their overall goals of a total of 120 minutes of sedentary behavior reduction and having 5 minutes of a sedentary break every 30 minutes by the end of the intervention.

To ensure consistent use of the Fitbit, participants will be instructed to wear the device during waking hours, including while showering or swimming. They will also be encouraged to wear the Fitbit at night, although this is optional. Participants are required to charge the Fitbit once a week, and we will send a text message every Sunday reminding them to charge and sync their Fitbit. We will track participants’ wear status over 12 weeks. If a participant fails to sync their Fitbit or wear it for two consecutive days, they will receive an automated reminder text message. If no improvement is observed within another two days, research staff will contact the participant by phone to troubleshoot issues.

#### 2.6.4. Smart water bottle

We will provide a smart water bottle (HidrateSpark, U.S.A) to each participant in the intervention group as a natural motivator to have sedentary breaks. The device alerts participants with a color change and message on the Fitbit to keep them hydrated. The device provides the daily water intake summary in the Fitbit app. The app calculates an individualized water intake goal based on the participant’s weight, age, and activity levels. By increasing water intake, participants will need to get up more frequently to use the bathroom and to refill their water bottles, which will break their prolonged sedentary behavior.

#### 2.6.5. Tailored text messages

Three text messages each week will be provided to support and enhance the habit formation process. Using data collected from the Fitbit device, participants will receive algorithm-derived, individually tailored text messages updating them on their weekly goal progress mid-week and a review of sedentary behavior at the end of the week. We will start each week on Monday and provide an end of week message on Monday morning. A midweek message will be sent on Thursday. Thursday’s message will provide midweek sedentary behavior feedback. Sunday’s message will encourage participants to achieve their goal and sync the Fitbit to the app. Monday morning’s (end-of-week) message will be structured by 1) algorithm-derived feedback (praise, reinforcement or encouragement messages), 2) a weekly summary of sedentary time, the longest sedentary period, and a suggestion for extra sitting breaks, 3) one tip from the TTT booklet, and 4) setting of next week’s goal on sitting hours, duration, and frequency of sitting breaks (Table 2).

**Table 2:** Examples of praise, reinforcement, and encouragement messages. The underline indicates personalized information.

Praise message If the participant achieves this week’s sedentary hours goal for > 5 days	Reinforcement message If the participant achieves this week’s sedentary hours goal for 3–5 days	Encouragement message If the participant achieves this week’s sedentary hours goal for < 3 days
Cheers to you on a job well done, Jane! You completed your daily sitting goal for 6 days. You spent 8.7 hours sitting daily. You could take extra breaks from sitting 19:00–19:45 on weekdays. Take a stand today! Challenge yourself to stand while talking on the phone.	You’re on the right track, Jane! You completed your daily sitting goal for 4 days during Week 1. You spent 9.8 hours sitting daily. You could take extra breaks from sitting 19:00–20:10 on weekdays. If you’re watching TV today, remember to get up and take a short walk during commercial breaks!	A little bit of effort goes a long way, Jane! You completed your daily sitting goal for 2 days. You spent 11 hours sitting daily. You could take extra breaks from sitting 19:00–20:40 on weekdays. A great way to break your sitting is to do some chores around the house. Meet your goals and get things done!

To develop and provide text messages, we will take the following steps.

**1. Creation of weekly text message template:** The team will create the weekly message templates for praise, encouragement, and reinforcement messages.
**2. Fitbit data transfer:** We will transfer the Fitbit data from the participants’ Fitbit accounts into REDCap on Thursday morning and Monday morning to provide Thursday (mid-week) and Monday (end-of-week) messages. There are several Fitbit data management platforms that summarize physical activity data, but none summarize sedentary behavior data. Therefore, the team developed our own data collection interface using Fitbit’s Web Application Programming Interface (API). This platform provides secure data acquisition and management tools that facilitate remote data collection from Fitbit. All Fitbit’s minute-level data will be imported into REDCap.
**3. Fitbit data aggregation and calculation:** Fitbit provides minute-level data of prelabeled “sedentary” time and step counts, as well as summary data for “light level”, “moderate”, and “vigorous” activity time, and does not provide meta-data shown in the Fitbit app (“longest stationary period” and “total stationary hours”), nor an algorithm to replicate the daily sedentary behavior meta-data. Therefore, we will reproduce the Fitbit app’s sedentary behavior meta-data. First, we will exclude days with less than 10 wearing hours [37] and remove the sleep period detected by Fitbit from calculation. The “total stationary hours” from Fitbit will be calculated by the sum of all sedentary minutes that had accompanying heart rate data during a selected day. The “longest stationary period” from Fitbit will be calculated by the longest uninterrupted period of the day at which zero steps were taken.
**4. Integration of Fitbit data into the text message template**: The team will take advantage of logics built into the REDCap environment to integrate information from the collected Fitbit data into our weekly text message template using the following algorithm. If the participant achieves the week’s sedentary hours goal for > 5 days, they will receive a praise message. If the participant achieves the week’s sedentary hours goal for 3–5 days, they will receive a reinforcement message. If the participant achieves the week’s sedentary hours goal for < 3 days, they will receive an encouragement message. The examples of praise, reinforcement, and encourage messages are provided in Table 2. The text messaging algorithm system has been used successfully before [38].
**5. Send text message by using Twilio and REDCap**: The customized text message will be sent to the respective participant using the Twilio plug-in for REDCap.
**6. Ask next week’s goals via text message**: This study will use REDCap’s function to administer a survey via SMS conversation to set a participant’s next week’s sedentary hours goal and numbers/frequency of sedentary breaks. Participants may respond to the SMS survey with any kind of alpha-numeric text. Participants’ answers on next week’s goals will be automatically saved into REDCap.

### 2.7. Control group

The control group will receive usual care and the American Heart Association (AHA)’s “Answers by Heart” fact sheets. Answers by Heart is a series of downloadable patient information sheets, which include information about monitoring body weight, blood pressure, and cholesterol, as well as general tips for cooking healthfully and engaging in physical activity [39].

### 2.8. Post-intervention visit

After 12 weeks of the intervention, all participants will be invited to an in-person visit at week 13. During the visit, all the above parameters will be re-measured by the same RA and all the above survey questionnaires will be completed. All participants will then wear the activPAL3 and the CGM for another 7 days to examine their post-intervention activity and glycemic control. They will return the devices via pre-paid mail. To enhance adherence to the protocol, all participants will receive $50 for the post-intervention visit, and an additional $50 for mailing the devices back to the study team (a total compensation = $150 per participant). A subsample of participants from the intervention group (n = 20) will be invited to participate in an exit interview.

### 2.9. Primary outcomes

#### 2.9.1. Feasibility and acceptability

Feasibility will be determined by analyzing dropout rates and intervention compliance. The dropout rate will be calculated as the percentage of participants who withdrew consent. Participants who drop out will explain their reasons. For compliance, we will confirm if over 75% of participants wore their Fitbit for at least 5 valid days each week (with a valid day being over 10 hours of wear) for 75% of the study duration (9 weeks), and if the Sit Less group responded to 80% of goal-setting texts. These criteria are derived from metrics used to assess the feasibility of consumer-based wearable physical activity trackers in other digital behavior change studies [40].

Acceptability will be assessed through two satisfaction questionnaires and exit interviews. Due to the absence of a standardized satisfaction questionnaire for multi-technology interventions, we merged two questionnaires to assess overall satisfaction with the Sit Less program and its individual components (Fitbit, Fitbit app, Fitbit’s Move Alert, smart water bottle, and text messages). Satisfaction with the overall program and each technology (Fitbit, Fitbit app, Fitbit’s Move Alert, smart water bottle) will be measured using modified items from Lyons et al.’s questionnaire [41]. Satisfaction with the overall program will be evaluated with six items like “I would recommend the program” and “The program is likely to reduce sedentary behavior.” Additionally, the perceived ease and continued intention to use each technology component will be evaluated with 11 items such as “I found the Fitbit/Fitbit app/Smart water bottle convenient to use.” and “I would continue to wear the Fitbit”. A separate questionnaire by Burner et al. [42] will assess the text messaging component, evaluating the relevance of the message contents to the participants, as well as the frequency, motivation, and timing of the messages. All responses will be made on a Likert-type scale from 1 (strongly disagree) to 5 (strongly agree). Our threshold for acceptability outcomes is established so that at least 75% of participants must either agree or strongly agree with each statement on the satisfaction questionnaires [42].

In addition to the satisfaction questionnaires, an exit interview (n = 20) will be conducted by the PI or trained research staff and audio-recorded. For the exit interview, we will develop a semi-structured interview guide and thematic analysis. The interview guide will be structured to capture additional feedback on the acceptability and satisfaction of the intervention, barriers and facilitators to sedentary behavior reduction, and perceived health impacts of sedentary behavior reduction.

#### 2.9.2. Changes of sedentary behavior

**Fig 4:**
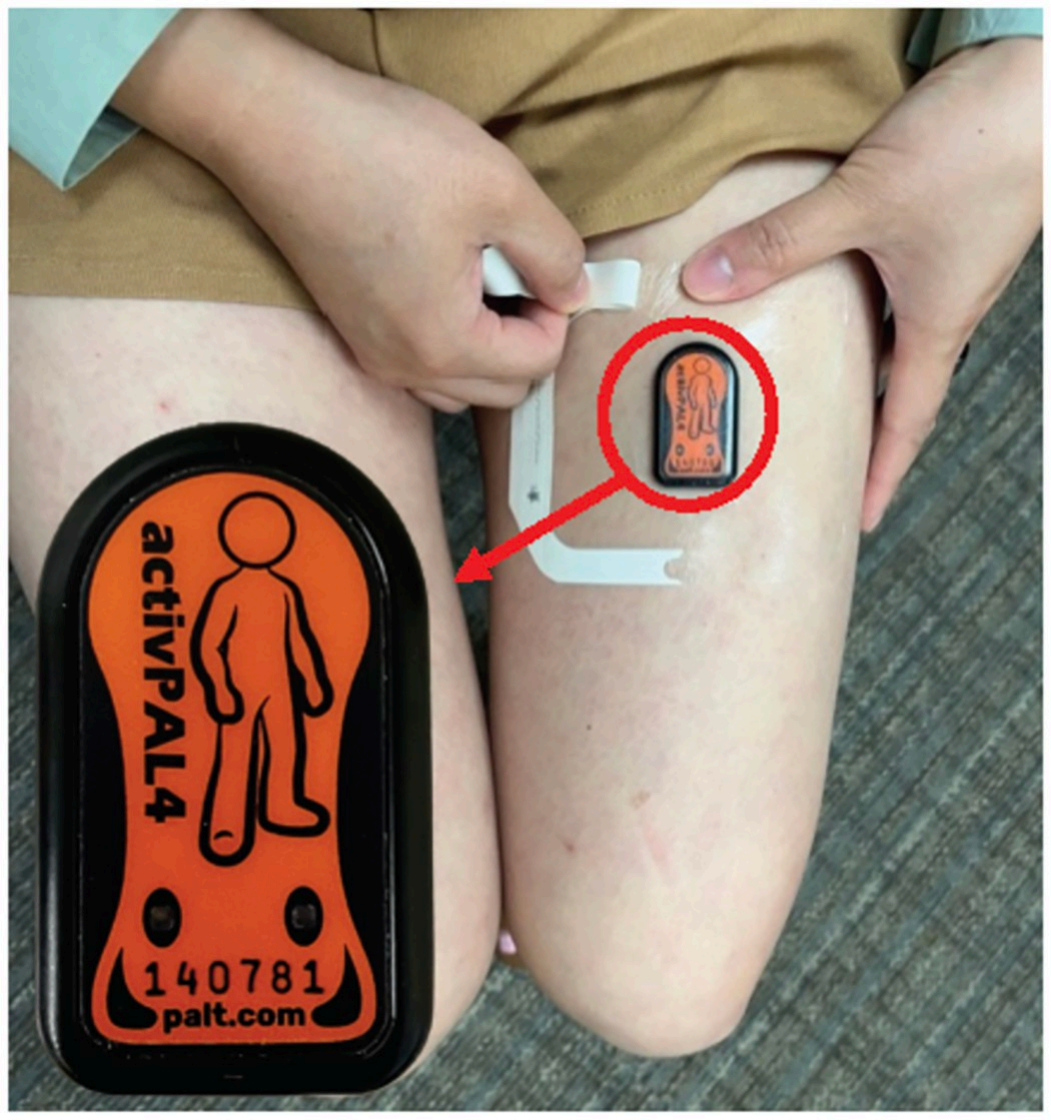
Participant wearing activPAL 3 on thigh for sedentary behavior and physical activity measurement.

Sedentary behavior will be assessed by three indicators including total daily sedentary time, prolonged sedentary time (time spent sitting >30 minutes and >60 minutes), and number of sit-to-stand transitions. These three sedentary behaviors will be measured by 7 days of activPAL 3 (PAL Technologies, U.K) device monitoring prior to the beginning of the intervention/control protocol and again after the conclusion of the protocol for both study arms. The activPAL 3 monitor is a tri-axial accelerometer that also includes an inclinometer, worn on thigh (Fig 4). Due to the activPAL 3 position on the thigh, the activPAL 3 monitor is the most sensitive device to distinguish sitting (horizontal position) and standing (vertical position) [43, 44]. The activPAL 3 has demonstrated great test-retest reliability and criterion validity with direct observation to classify sitting, standing, and stepping [45].

### 2.10. Secondary outcomes

#### 2.10.1. Physical activity

Time spent standing and walking (stepping), number of sitting-to-standing transitions, and number of step counts will be measured using the activPAL 3 device. The activPAL 3 has demonstrated greater criterion validity compared to video observation for measuring physical activity [46].

#### 2.10.2. Cardiometabolic markers

Height, weight, and waist and hip circumferences will be measured using a validated stadiometer, digital scale, and flexible tape measure, respectively. All measurements will be recorded to the nearest 0.1 cm and 0.1 kg. Blood pressure will be measured using an automated and validated blood pressure monitor. The device will take three readings at 2-minute intervals and the mean of three BP readings will be recorded. For blood samples, participants will be required to fast overnight (at least 8 hours). Cardiometabolic markers including fasting lipids, insulin levels, HbA1c, and hs-CRP levels will be collected via finger prick by a trained research staff member, using dried blood spot cards for measurement and analysis at ZRT Laboratory (Beaverton, OR, U.S.A). Lastly, 24-hour glucose levels will be measured by the use of a continuous glucose monitor (Freestyle Libre 2, Abbott, U.S.A) over a 7-day period at baseline and post-intervention. The 24-hour glucose control will be evaluated by mean 24-hour glucose levels, glucose management indicator, glycemic variability (%), % time below range (glucose < 70 mg/dL), % time in range (glucose 70–180 mg/dL), and % time above range (glucose >180 mg/dL) [47].

#### 2.10.3. Patient-centered outcomes

Confidence in reducing sedentary behavior will be measured using the 12 items from the Self-Efficacy Questionnaire for Physical Activity and Sedentary Behavior (previously reported Cronbach’s α = 0.79) [48]. The items assess the level of confidence for specific sitting behaviors and sedentary breaks. Habit strength for sedentary behavior will be assessed by using the validated Self-Report Habit Index measure (previously reported Cronbach’s α = 0.91) [49]. This 7-item index was adapted to assess the degree to which sedentary breaks (standing/walking) become habitual [49]. Fear of movement will be measured by using the Fear Avoidance Belief Questionnaire. The questionnaire consists of 5 items regarding patients’ beliefs about the relationship between perceived discomfort and movement, which can lead to fear of movement and avoidance of physical activity/exercise. The words “pain” and “back” were changed to “heart discomfort” and “heart” respectively, as used by Ahlund et al. [50]. The questionnaire was also validated for patients with CVD. Depressive symptoms will be measured using the Patient Health Questionnaire 9 (PHQ-9) [51].

### 2.11. Demographic characteristics and potential covariates

We will collect standard demographics, socioeconomic characteristics, medical history, and current medication via self-report. We will also ask about current occupation activity, which ranges from “mostly sedentary” (e.g., desk-based job) to “very active and physical” (e.g., manual labor). Participants will answer questions about their tobacco use, alcohol intake, dietary habits as measured by the 16-item Rapid Eating and Activity Assessment for Participants Short Version (REAP-S) [52], and sleep quality as measured by the 10-item Pittsburgh Sleep Quality Index (PSQI) [53].

### 2.12. Data management plan

Study participants will complete questionnaires online via REDCap, and the study team will review the responses in the presence of the participants to clarify or complete any missing information. REDCap, a secure, HIPAA-compliant web-based application developed at Vanderbilt University, will be used to manage all data. It provides data entry, audit trails, and export procedures, and flags inconsistent or out-of-range responses for review.

### 2.13. Analysis plan

Feasibility (dropout rates and intervention compliance) and acceptability outcomes (assessed by satisfaction questionnaires) will be calculated and reported as percentages. The determination of whether the Sit Less program is feasible and acceptable will be based on the previously defined criteria. The recorded exit interview will be transcribed verbatim and analyzed using a thematic approach to guide refinements to future sedentary behavior reduction interventions as well as to identify barriers and facilitators to sedentary behavior reduction. To enhance credibility, we will follow the consolidated criteria for reporting qualitative research (COREQ) guidelines [54].

For sedentary behavior and light PA data from the activPAL 3, we will only include data of daily wear time >10 hours, with a minimum of 4 valid days [37]. The activPAL data will be downloaded and analyzed by using the activPAL 3 processing software and applying “CREA (enhanced analysis algorithm)” processing setting (PAL analysis, V8.11, PAL Technologies). The CREA algorithm demonstrated excellent agreement with diary data [55] and strong correlation with using a traditional algorithm to assess wake time activities [56].

Once all the data from activPAL 3 have been processed, statistical analyses will be conducted using STATA 18 (StataCorp, U.S.A). All analyses will adhere to an intention-to-treat approach, and there will be no imputation of data. Given this simple pre-post design, participants in both study groups without a post-intervention value for a respective outcome measure will not be included in the analysis of effects on that measure. Demographic and other baseline values will be compared between those who do and do not complete the study to inform potential modifications to the intervention for future studies. Descriptive statistics will summarize the demographic and clinical characteristics of the participants. Chi-Square, Mann-Whitney or independent *t*-tests will be used to inform potential differences between control and intervention groups at baseline. We will consider sex as a biological variable and conduct subgroup analyses to determine if there are differences in sedentary behavior outcomes and the acceptability of the Sit Less program.

We will use generalized linear models to assess the effects of the intervention on the key study outcomes. We anticipate that most will use the Gaussian family of distributions with the log link function, yet some are likely to be severely skewed or demonstrate a better fit using the Poisson family or nonparametric regression models. Estimated mean differences in the post-intervention values controlling for the baseline assessments will be generated from each respective outcome model. These estimated adjusted mean differences will be used to evaluate the effect of the study group and to serve as measures of effect sizes. For example, if the Sit Less group sits for 60 minutes less than the control group after the intervention, this is crucial to comprehending the effects of the intervention and to inform the design of future large-scale RCTs. Furthermore, we will generate bootstrapped 95% confidence intervals around each adjusted mean difference. All models and estimations of adjusted mean differences will use the robust or sandwich estimator of variance. While we will consider results statistically significant if p < 0.05, we will not adjust this critical alpha level for the multiple outcomes assessed in this work, as the focus is on the estimated mean differences for informing future work.

### 2.14. Oversight and monitoring

#### 2.14.1 Data monitoring

As a single-site, low-risk behavioral study, we will not establish a Data Safety and Monitoring Board, nor conduct interim analyses. Instead, the PI (CP), supervised by the coauthor (SM) and aided by the study team, will bi-monthly monitor enrollment, data quality, adverse events, and participant dropout rates. Data from the continuous glucose monitoring and activPal devices will be assessed for validity immediately after return. Should data quality be below standards, the team will review the monitoring protocols and measures will be re-taken accordingly.

#### 2.14.2. Harms and auditing

The study team, along with the PI, will hold weekly meetings to assess the progress of the study, and review any potential problems or adverse events. Expected adverse events include muscle soreness, discomfort from frequent sedentary breaks, skin irritation from the activPAL and Fitbit, and local redness, inflammation, or bleeding at the CGM sensor site. Serious and unexpected adverse events related to the study protocol will be reported to the IRB within seven days. Annually, the study will undergo continuing review and be granted approval by the IRB.

### 2.15. Ethics and dissemination

#### 2.15.1. Ethics

The research study complies with the Declaration of Helsinki and has been reviewed and approved by the Vanderbilt University Medical Center IRB (#220416). Written informed consent will be obtained from all subjects involved in the study with the assistance of the trained research staff. Any necessary protocol amendment will undergo IRB approval and be reflected in the clinical trial registry.

#### 2.15.2. Confidentiality

Participant confidentiality is ensured by coding personal information with a four-digit number and storing participant identifiers separately from research data. An anonymous email account will be used for the Fitbit and HidrateSpark apps to maintain anonymity. All electronic records will be password-protected and accessible only to authorized team members, with paper documents secured in the VUSN Research Clinic. After seven years post-study, all identifying data will be destroyed.

#### 2.15.3. Dissemination plan

When we complete the analyses of data, the data will be accessible through a data repository. We will disseminate the study findings by publishing the results in scientific journals and all participants will be provided with a summary of the findings.

## Discussion

The study aims to evaluate a multi-technology-based and individually tailored intervention to reduce sedentary behavior in CVD patients. The primary outcomes are the feasibility, acceptability, and primary efficacy in changing sedentary behavior (change in total minutes spent in sedentary behavior, change in prolonged sedentary time, and number of sit-to-stand transitions) measured by the activPAL. Secondary outcomes are changes in physical activity measured by the activPAL and changes in cardiometabolic biomarkers measured by CGM and dried blood spot cards, along with changes in psychosocial constructs.

Despite the important role of sedentary behavior in cardiac outcomes in CVD patients, there are few studies focusing on reducing sedentary behavior in this understudied population. In this study, we will test a sedentary behavior reduction intervention program based on habit theory that leverages wearable technology, for CVD patients. The proposed sedentary behavior reduction program is innovative because it side-steps barriers of exercise (e.g., fear of heart discomfort, fatigue) in CVD patients and focuses on breaking sedentary time with short bouts of standing and walking, which can be easily implemented in the natural environment. Other novel aspects of this study include using multiple technologies, including a Fitbit and a smart water bottle to motivate participants to stand up, and the development of an automatic, yet tailored text message algorithm. The study will also employ a validated measure for sedentary behavior (i.e., activPAL) and sensitive biological indicators linked to sedentary behavior, such as 24-hour glucose monitoring parameters.

However, there are also several limitations and concerns. First, our ultimate goal is to disrupt prolonged sedentary behavior by encouraging movement every 30 minutes. However, there’s a limitation with the Fitbit device as it only sends alerts after one hour of inactivity. To address this issue, we will send text messages and provide tips that assist participants in gradually transitioning from breaking sedentary behavior every hour to every 30 minutes. Once they have successfully established the new habit with the aid of their Fitbit’s one hour alert, we will then motivate them to progress towards breaking their sedentary behavior every 30 minutes. Another concern is the risk of cross-contamination between different arms of the study, a result of enrolling subjects from a single hospital. The applicability of the findings may also be limited when considering a wider demographic, such as urban populations with access to extensive public transportation systems. Lastly, this study does not address other lifestyle factors tied to CVD management, such as dietary habits. However, the primary focus is to establish the efficacy of this intervention in reducing sedentary behavior before addressing other lifestyle factors.

## Conclusions

Though people with CVD are particularly prone to high levels of sedentary behavior and prolonged sedentary behavior, there are few interventions targeting sedentary behavior in people with CVD. Implementing frequent but short periods of sedentary breaks (standing or walking) in daily life can be attainable behavioral goals that can reduce total sedentary time and yield substantive health improvements in people with CVD. In our pilot randomized control trial, we will develop a multi-technology-based intervention to decrease sedentary behavior among CVD patients. This intervention will utilize a Fitbit device with sedentary break reminders, a smart water bottle, and personalized weekly text messages for a sample of 70 individuals with CVD. The study will determine if a low-touch, home-based mHealth intervention is feasible, acceptable, and effective for CVD patients in decreasing their sedentary behavior. The results could guide the development and implementation of future studies targeting sedentary behavior in a broader population, ultimately contributing to the prevention of CVD complications and the enhancement of cardiometabolic health.

## Supporting information

S1 Checklist SPIRIT checklist. (DOC)

S1 File (PDF)
