# Supplementary material for: A multiple technology-based and individually-tailored Sit Less program for people with cardiovascular disease: A randomized controlled trial study protocol
Source: PLoS One. 2024 May 9;19(5):e0302582. doi: 10.1371/journal.pone.0302582 (PMC11081313; doi:10.1371/journal.pone.0302582)
Supplement: S1 File — (PDF) [file pone.0302582.s002.pdf]

Study Type and Performance Site Information

Type of study:
[x] **Standard or Expedited**
[ ] Exempt
[ ] Umbrella Review for funds release
[ ] Non-Human Subject Determination
[ ] Quality Improvement/Non-Research Determination
[ ] Request review by another IRB
[ ] Coordinating Center ONLY

Please indicate which Committee is most appropriate to review your project:
[x] **Social and Behavioral Sciences**
[ ] Health Sciences

Are there any international sites involved in this study in which the PI is responsible?
[ ] Yes
[x] **No**

Is this project cancer-related?
[ ] Yes
[x] **No**

Study Purpose and Description

Provide a brief abstract of the study in lay language. The IRB Committees are comprised of scientists with varied backgrounds, non-scientists, and community members.
Too much sitting may cause heart problems. In particular, people who have cardiovascular disease tend to sit 10-14 hours a day, which is most of their waking time. Exercising may lessen some of the negative effects of too much sitting. However, many heart patients do not exercise enough. We need to find new ways to reduce sitting and improve physical activity. One easy way is breaking up long periods of sitting with a short period of standing or walking.
In this study, we will develop an intervention for cardiovascular patients to sit less and move more (Sit Less Program) The intervention group (35 patients) will receive a Fitbit and turn on the "move" alert on the watch. This alert will help them to stand and move frequently throughout the day. They will also receive a HidrateSpark Smart Water bottle to keep them hydrated and break their sitting via frequent urination. They will attend one instruction/goal setting meeting and receive three weekly text message to set a goal and check their progress. This will last for 12 weeks. The control group (the other 35 heart patients) will receive an American Heart Association's healthy living booklet.
We will examine whether our intervention can help cardiovascular disease patients break up their sitting, reduce daily sitting time, and move more. We will also study whether the program leads to improvements in heart disease risk factors, and whether cardiovascular disease patients like the program and can follow it. Breaking long sitting with only a few minutes of standing and walking throughout the day is simple and easy. If we find that this program is acceptable to heart patients and reduces their sitting time, it may help improve their heart outcomes in the future.

Expected duration of the study.
2 years

The IRB needs to understand how this study adds to the knowledge on this topic in order to be able to judge the risks and benefits to the research participants.
Sedentary behavior (SB) is a strong modifiable risk factor for cardiovascular disease. During SB, there is no muscle contraction of the legs, which causes decreasing insulin sensitivity, vascular dysfunction and promotes activation of low-grade inflammatory cascades. As a result, greater total SB time is related to increased cardiovascular disease (CVD) risk. For example, greater total SB time is related to decreased high-density lipoprotein (HDL) and increased triglycerides (TG), fasting glucose, BMI, and waist circumference (WC). Further, SB time is related to increased risk of CVD incidence; one hour increase in objectively measured SB time was associated with a 12% increase in incidental CVD regardless of physical activity (PA) levels. Along with the total SB time, SB accumulation patterns are also associated with CVD outcomes. People sitting for a long time without interruption (prolonged patterns) have poorer cardiometabolic outcomes than people with interrupted sitting patterns.

Despite the important role of SB in CVD, patients with CVD remain highly sedentary and have prolonged patterns; they spend 10-14 hours per day in SB, which comprises 70-90% of waking time, and 50% of their total SB time is prolonged (>30 minutes per bout). Some (10-21%) CVD patients engage in recommended levels of moderate-to-vigorous physical activity (MVPA), but their waking time is mostly occupied by SB. Emerging evidence suggests that PA attenuates CVD risk, but does not eliminate the increased risk associated with high SB time.Considering CVD patients' low cardiorespiratory fitness and low activity levels, they may be at greater risk than others for the negative cardiovascular consequences of SB. Therefore, there is a pressing need to develop novel strategies to reduce SB and improve daily activity in patients with CVD. Targeting SB, which occupies most of a CVD patient's waking time, can be a new target behavior for secondary prevention in this population.

Research, Activities, Procedures, and Schedule of Events for Study Participants

Please check all that apply to your study and describe each below.
[ ] Behavioral Observation
[x] **Randomization**
[x] **Blinding**
[x] **Surveys, Interviews, Questionnaires**
[ ] Document and Artifact Collection
[x] **Deception, Withholding or Postponing Medications/Treatments, or Imposing other Restrictions**
[x] **Audio/Video Recording**
[ ] Sham Procedure
[x] **Specimen/Data Collection and/or Storage**

DATA COLLECTION, STORAGE OF DATA/SPECIMENS, AND/OR ISSUES OF CONFIDENTIALITY - Describe the procedures that will be utilized to protect the privacy of the research participant. Include who will have access to the research information (for example, video/audio recordings, discovering information about the participant that could be harmful if released such as mental illness, genetic information, sexual preference, drug abuse, etc.) and where it will be stored.
To protect the privacy of our participants, informed consent procedures will be conducted in a private testing room. The questionnaires will be completed by the participant with assistance from a trained research staff member. REDCap will be utilized and participants will select their answers on the secured iPad. The responses will be saved in the secured REDcap server.

After completing the surveys, participants will wear the activPAL3 device (PAL Technologies Ltd., Scotland, UK) and a continuous glucose monitoring device (CGM, FreeStyle Libre, Abbott) for 7 days. The devices will be attached by the participant in a private room to protect their privacy. The electronic data from activPAL 3 and continuous glucose monitoring devices will be downloaded by the trained research staff and saved into a password-protected computer at a locked office. Also, a daily sleep diary and wear log will be distributed, a one-page document, and the returned log will be stored in a locked filing cabinet at a locked office.

The trained research staff member will also complete baseline biometric assessments, including measurements of height, weight, waist circumference, blood pressure, and blood collection from a finger prick (8-9 drops). This biometric assessment will be conducted in a private room and these data will be recorded in REDCap. The dried blood spot cards will be kept in a locked cabinet at a locked office.

A subsample of participants from the intervention group will be invited to participate in a separate qualitative exit interview. The exit interview will be conducted by a trained study team member using the interview guide during the visit. The audio recordings will be recorded in a secured iPad and transcribed verbatim. The audio recorded files and transcripts will be saved in the secured computer at a locked office.

As a part of the intervention, the study team will collect the intervention group's Fitbit data. To ensure participants' anonymity, confidentiality, and privacy, the research staff will (1) set up anonymous Gmail accounts and (2) set up de-identified Fitbit accounts for the purpose of the study. We will not use their real name or email address, and will default their account settings to the most privacy-protective option available. Research staff and participants will be advised that the email and Fitbit accounts should be used strictly for the study's purpose. Account information will be stored in the protected PI's computer within encrypted documents. Fitbit data will be saved into REDCap and only accessible by the PI and/or the IT team.

As another component of the intervention, we will send weekly text messages to the intervention group. The text messages will be sent from REDCap via the third-party service Twilio.com. Participants' text replies do not get permanently logged on Twilio's servers but instead remain securely and separately in REDCap. Twilio configuration is managed by a REDCap administrator.

The PI and staff have been trained in confidentiality and HIPAA requirements. Data analyses will be completed using de-identified data.

Describe how the confidentiality of participants' data will be assured. Include a description of any issues specific to the study that might increase the risk of breach of confidentiality. Describe how codes will be generated if codes are used to protect identities, and who will have access to such codes. If a certificate of confidentiality will be provided, include the name of the person holding the certificate. Describe the final disposition of research data when the study is concluded (e.g., will information be destroyed, will the PI maintain the information indefinitely, etc.).
All Personal Health Information (PHI) and Personal Identifying Information (PII) will be kept confidential, unless release is required by law. Release of PHI/PII information will only be allowed if it is legally required by law. The PI and staff have been trained in confidentiality and HIPAA requirements and will conduct the study using Good Clinical Practice guidelines.

Each subject's information will be coded with four digit numbers (ex. 0001) at the time of entry into the research study so that no direct identifiers appear on any samples or questionnaires. We will also create an anonymous email account to use for the Fitbit and HidrateSpark apps. The study ID and the anonymous email will serve as the only identifiers used on all study-related documents including Fitbit data, continuous glucose monitoring data, questionnaires, activPAL data, sleep and wear logs, and interview recording files. Personal or private identifiable data will not be stored on portable devices. The code identity and anonymous email account information will be stored in the master list with full name and contact information (e.g., phone number, physical address, email address). The master list will be used for administrative purposes only and accessible to and maintained by approved study staff and PI. The master list will be stored in a file separate from the coded study dataset in an encrypted file on a password-protected computer.

Hard copy data (sleep and wear logs) and other study documents and specimens (dried blood spot cards) will be kept in a locked file cabinet in a safe location. Dried blood spot cards do not need refrigeration. Dried blood spot cards will be sent to the ZRT company for analysis and will be disposed of by the ZRT laboratory. The test results will be received via the secured ZRT laboratory's email system.

All electronic records, including the master list, voice recordings, activPAL, and continuous glucose monitoring data, will be stored on a secure computer at Vanderbilt School of Nursing and password-protected, only accessible by the PI and/or her staff. Two e-consents (prescreening consent and main consent), survey data, and Fitbit data collected using the Internet will be collected via REDCap, a HIPAA compliant platform. No identifiable information will be stored on any mobile devices (laptops, USB keys, CDs, DVDs, etc.). After a period of five years from the end date of the study, all identifying information, including signed consent forms and the master list, will be destroyed. Anonymous raw data and electronic data will be maintained indefinitely.

Data entry will be completed in REDCap by the PI and/or her staff and will be imported to SPSS file format for analysis. Only de-identified data will be used for analysis.

RANDOMIZATION - Describe the randomization process (who will randomize, how will randomization be determined, etc.)
Once baseline assessment is completed, the PI will use the REDCap randomization function and will inform participants of their group assignment.

BLINDING - Describe who will be blinded and if/when research results or previously blinded treatment assignments will be made available to participants. Include the provisions for breaking the blind (e.g., emergency situations, participant's request, etc.).
Participants and the PI will be aware of the participants' treatment assignments. However, data collectors will be blinded.

SURVEYS, INTERVIEWS, AND QUESTIONNAIRES (please see Help for important information on payments to participants) - If surveys, interviews or questionnaires will be used as part of this study, indicate who will conduct the survey, interview or questionnaire and his/her qualifications. In addition, describe the setting and mode of administering the instrument (e.g., by telephone, one-on-one, group, etc.) and attach a copy of the instrument.
At the Baseline visit 1, those who are consented will participate in completing survey questionnaires. The questionnaires will be completed by the participant with assistance from a trained research staff member. REDCap will be utilized and participants will select their answers on the secured iPad. The responses will be saved into the secured REDcap server. The research staff will provide one page of the daily sleep diary and wear log and ask participants to complete it every day for 7 days while they are wearing the activPAL device. The survey will be stored in a locked filing cabinet and office.

After 12 weeks of intervention, all participants will be invited to an in-person visit (post-intervention visit). During the visit, all the above surveys will be re-administrated with assistance from the trained research staff. A subsample of participants from the intervention group will be invited to participate in a separate qualitative exit interview. The exit interview will be conducted by a trained study team member who has experience in conducting an interview. The study team developed the interview guide and will do rehearsal to ensure the reliability. The interview will last 30 minutes and be audio recorded in a secured iPad and transcribed verbatim. The audio recorded files and transcripts will be saved in the secured computer.

DECEPTION, WITHHOLDING OR POSTPONING MEDICATIONS/TREATMENTS, OR IMPOSING OTHER RESTRICTIONS - Describe the methods of deception to be used, the medications being withheld or postponed, the length of time medications will be withheld or postponed, any other restrictions to be imposed on participants (i.e., diet, exercise), and the precautions taken to decrease or eliminate risks to participants.
The participants will be asked to fast from midnight (at least 8 hours) for the dried blood spot test for cardiometabolic profiles (lipids, insulin, glucose, HbA1C, and CRP). However, we will not ask them to withhold or postpone their medications. After obtaining blood samples, we will provide snacks and drinks.

AUDIO/VIDEO RECORDING - Describe how the audio/video recordings will be stored, as well as how they will be disposed of when this research is complete. Describe how the participant's confidentiality will be maintained.
After the intervention, an exit-interview will be conducted with a subsample of participants from the intervention group who are willing to do the interview. The interview will be recorded by using a secured iPad and the recording files will be saved in the secure computer at Vanderbilt School of Nursing. Only the PI and study staff will assess the files. When the recordings are transcribed, identifiable information will be deleted. The recording files will be permanently disposed 5 years after the study has been completed.

SPECIMEN COLLECTION - Describe all procedures used for specimen collection.
Participants will be required to fast overnight (at least 8 hours) prior to the study visit. Blood samples will be collected via finger prick by a trained research staff member. The 8-9 drops of blood will be dropped on a dried blood spot card. The collection will occur at the Vanderbilt School of Nursing clinic located at the Wesley Building. After the blood collection, the dried blood spot card will be stored in a locked cabinet (no need to be refrigerated) at the Vanderbilt School of Nursing clinic. The dried blood spot card will be wrapped using supplies from the ZRT laboratory and sent to the lab via mail. The dried blood spot card will be analyzed at the ZRT laboratory to test for fasting glucose, insulin, hsCRP, HbA1c, and lipids.

Will you be performing a blood draw(s)?
[ ] Yes
[**x**] **No**

Will specimens be obtained for genetic testing?
[ ] Yes
[**x**] **No**

Will the PI create a repository at VU/VUMC with any of the specimens and/or data for future use?
[ ] Yes
[**x**] **No**

Please indicate all procedures and activities performed for research purposes only and the frequency at which they occur in the study (e.g., skin biopsy, 3 times).

Procedure/Activity	Frequency
Surveys	2 times
Physical activity monitoring (ActivPAL) for 7 days	2 times
Continous glucose monitoring (Freestyle Libre) for 7days	2 times
Physical exam (anthropometric measures, blood presure)	2 times
Blood collection via finger prick (dried blood spot card)	2 times
Exit interview	1 time
7 days of sleep diary and wear logs	2 times

If all of your study is minimal risk, please indicate the categories that it fits 45 CFR 46.110 or 21 CFR 56.110:
[ ] N/A: Study is greater than minimal risk or Standard
[ ] (F)(1) Drugs or devices where no IND/IDE is required
[**x**] **(F)(2) Collection of blood by stick or venipuncture**
[ ] (F)(3) Prospective collection of specimens by non invasive means
[ ] (F)(4) Collection of noninvasive data through routine clinical practice
[ ] (F)(5) Research on materials that have been collected for non research
[**x**] **(F)(6) Collection of data from voice, video, digital or image recordings**
[**x**] **(F)(7) Research on individual or group characteristics (surveys)**

Please indicate the type of data collected.
Self-reported surveys, finger-prick based blood collection, 7 days of physical activity, 7 days of continuous glucose levels, 7 days of sleep diary and wear logs, audio recordings (from interview)

Please describe type of survey or focus group, evaluation methods used.
At Baseline, participants will be given an iPad to answer questions about their demographics, socioeconomic characteristics, and a series of questionnaires detailed below. All responses will be recorded in REDCap.
a) Demographics: demographics and socio-economic status will be self-reported by participants.
b) Medical history and current medication: Medical history and current medication will be self-reported by participants.
c) Health behavior: Questions about their tobacco use, alcohol intake, physical activity (measured by the 16-item Global physical activity questionnaire), sedentary behavior (measured by the11-item Weekly sitting inventory), diet (measured by the 16-item Rapid Eating and Activity Assessment for Participants Short Version (REAP-S), and sleep quality (measured by the Pittsburg sleep quality index) will be answered with the assistance of the study team member.
c) Fear of movement: Fear of movement will be used by using the Fear Avoidance Belief Questionnaire. The questionnaire consists of 5 items regarding patients' beliefs about the relationship between perceived discomfort and movement, which can lead to fear of movement and avoidance of physical activity/exercise. The words "pain" and "back" were changed to "heart discomfort " and "heart", respectively as used by Kristina Åhlund et al. The questionnaire was also validated for patients with myocardial infarction.
d) Confidence in reducing SB and increasing PA: Confidence in reducing SB and increasing physical activity will be measured using 12 items from the Self-Efficacy Questionnaire for Physical Activity and Sedentary Behavior (Cronbach's alpha = 0.79).
e) Habit strength for SB: Habit strength for SB will be assessed by using a validated measure, Self-Report Habit Index (Cronbach's alpha = 0.91). This 7-item index was adapted to sedentary breaks (standing/walking) to assess the degree to which sedentary breaks become habitual.
f) Quality of life: Quality of life will be measured by using the 12-item Short Form Health Survey (SF-12). The SF-12 is well-validated in T2D patients and provides physical and mental health scores.
g) Medical history and current medication: Medical history and current medication will be self-reported by the patients.
h) Depressive symptoms: Depressive symptoms will be measured by using Patients Health Questionnaire 9 (PHQ-9).

At post-intervention, participants will answer the same surveys listed above. In addition, for the intervention group only, participants' level of satisfaction with the intervention will be assessed by using 23 items from the questionnaire developed by Lyons et al. and Burner et al.

Data and Safety

Describe how the risks to participants are minimized (e.g., screening to assure appropriate selection of participants, identify standard of care procedures, sound research design, safety monitoring and reporting).
Consent
For the pre-screening activity, potential participants recruited from the email distribution list or ResearchMatch will sign an e-consent in REDCap. Participants will be given information about what questions we will ask. There are no benefits or side effects related to this prescreening activity. Once they meet all of the eligibility criteria, they will be invited for the in-person baseline visit. At the visit, participants are given as much time as they would like to consider participation from the initial invitation to participate until they sign the "main" consent form. Participants are encouraged to ask as many questions as possible and reminded that participation is completely voluntary and will not affect their medical rights. They are also told that they have the option to discontinue participation at any time for any reason.

Finger Prick Blood Collection
Risks associated with the finger prick include: temporary discomfort from the needle prick, bruising, and rarely (< 1%) infection. These risks will be minimized by using a sterile technique and applying sustained pressure to the site.

ActivPAL
The activPAL activity tracker is a safe, non-invasive method to capture information about activity cycles. Participation in this study requires that participants wear an activPAL tracker taped (using Tegaderm adhesive dressing) to the front of the right thigh during waking hours for a minimum of 7 consecutive days (baseline and follow-up). The Tegaderm adhesive dressing used to apply the activPAL devices are hypoallergenic and consist of a dual layer hydrogel that does not pull at the skin or hair. They are used for waking day recordings and are removed before going to bed, showering or bathing and will therefore be reapplied each morning. There are few risks associated with wearing the activPAL device and include slight discomfort, such as light pressure from the activPAL or irritation from the waterproof dressing.

Continuous Glucose Monitor
The FreeStyle Libre Pro Flash Glucose Monitoring System is a FDA-approved, professional continuous glucose monitoring device indicated for detecting trends and tracking patterns in persons (age 18 and older) with diabetes. After application of the sensor, only a thin, flexible filament will remain under the skin and the sensor is held in place by an adhesive. The following are possible adverse effects of inserting a sensor and wearing the adhesive patch: local erythema (redness), local infection, inflammation, pain or discomfort, bleeding at the glucose sensor insertion site, bruising, itching, scarring or skin discoloration, and adhesive irritation. There is a remote risk of sensor or needle fracture during insertion, wear or removal, with fragments retained under the skin.

Fitbit
Participants will wear an additional wrist-worn activity tracker for 12 weeks during waking hours. There are few risks associated with wearing the device and include slight discomfort, such as light pressure from the wristband or irritation from wearing a damp band after showering or swimming. There is a possible risk of confidentiality loss. To reduce the risk, we will create an anonymous email account for the Fitbit app. All identifiable data will be kept on password-protected computer systems in a locked office.

Smart Water bottle
Participants will use smart water bottle to increase the frequency of standing and moving by frequently going to the restroom and kitchen for refill. There is no risk associated with using the smart water bottle. To reduce a possible risk of confidentiality loss, we will create an anonymous email account for the HidraSpark app. All identifiable data

will be kept on password-protected computer systems in a locked office.

12-Week Sedentary Behavior Intervention
Participants will gradually decrease their sedentary behavior and replace the sedentary behavior with a short period of light intensity physical activity such as 2 to 3 minutes of standing or walking over 12 weeks. The risk of falling or injury is the same as would be associated with day-to-day activity. Study staff members will instruct participants to make sure there are no hazards within the walking path and surrounding area.

Suicidal ideation or severe distress
Research has confirmed that simply asking a participant about whether they have thoughts of suicide is not a likely trigger of such an event43. It is usually only when the person reports ideation as well as an intent, plan, and/or means to commit suicide that risk for immediate suicide is considered to be more acute.

If a participant reports severe distress or suicidal ideation during the survey administration (Baseline visit 2 or Post-intervention visit), the research staff will immediately notify it to the PI. The PI will provide the participant with help to get treatment. This may include:
• working with the participant to contact his/her doctor,
• contact a trusted family member, or a therapist to discuss his/her thoughts,
• or work with the participant on a plan that may include getting him/her to a hospital for safety.

Describe how the risks to participants are reasonable in relation to anticipated benefits (e.g., includes benefits to the individual as well as to human kind, indicate how the risks are justified in this population).
The potential benefit of this study is to have an opportunity to lose weight, learn new tips about reducing sitting time and moving more. Participants will gain knowledge about their sitting patterns, physical activity patterns, and heart-related biomarkers.The results of this study will identify processes that are critical to the success of a future larger randomized trial including: a) recruitment and retention rates, b) refusal rates, c) sample size estimates, and d) effective intervention strategies and components. This knowledge may help develop a new physical activity and sedentary behavior guideline for heart patients and provide evidence about developing a Fitbit-based sedentary behavior reduction program for this population. Collectively, there are more benefits than risks.

Is there a data safety monitor or board/committee to review this study for safety and adherence to the study protocol?
[**x**] **Yes**
[ ] No

Describe the composition of the committee and their qualifications.
An independent Data and Safety Monitoring Board (DSMB) of 3 individuals not affiliated with the study will be convened by the PI, Dr. Park. Although not identified at this time, members of the DSMB board will include a VUSN research faculty member and a biostatistician.

Provide a general description of the data and safety monitoring plan.
PI, Dr. Park, RN, PhD will act as the internal study monitor. During the 2-3-month start-up phase, the entire research team will have weekly meetings. All research staff will complete training in Human Subjects Research (CITI courses), focusing on the modules for Social/Behavioral research and Good Clinical Practice. After the start-up meetings, meetings will be bi-monthly during the duration of the study. Dr. Park will train the research staff who will conduct data collection and exit interviews. Dr. Park will monitor subject enrollment, questionnaire, physical activity and continuous glucose monitoring, and specimen collection. The items to be monitored include but are not limited to: attrition rate including reasons for subject drop-out, adverse events, unanticipated problems, and data quality.

Describe plans for monitoring the progress of trials and the safety of participants (e.g., timing of DSM reviews and reports, planned interim analysis, etc.).
Meetings will be convened in person, at a minimum, every 6 months, and more often by teleconference if indicated or requested by DSMB committee members. We do not plan to perform efficacy interim analyses because the relatively small sample size and the number of primary outcomes will make it unlikely that we will have sufficient data at interim time periods to be interpretable in relation to stopping or modifying the study. However, we will rigorously and carefully monitor adverse events. The interim analysis reports for the DSMB will include but will not be limited to such tabulations as: 1) actual recruitment, versus planned recruitment and 2) adverse events.

Describe plans for assuring compliance with requirements regarding the reporting of adverse events (AEs), including plans for reporting of AEs to the IRB and appropriate regulatory agencies.
All adverse events will be reported in compliance with IRB policies and procedures. These adverse events include any serious adverse event that in the investigator's opinion was unanticipated or unexpected, involved risk to participants or others and was possibly related to the research procedures; and/or any noncompliance with the IRB-approved protocol that increased risk or affected the participant's rights, safety, or welfare. The research staff will notify the PI of any anticipated problems/adverse events immediately upon discovery. The PI will consult and determine the severity and appropriate response or action to the reported adverse event. All adverse events (which may include breach of confidentiality) which are serious, unanticipated, and related to the research procedures will be reported to the IRB within 7 calendar days of the PI's knowledge of the event.

Describe plans for assuring that any action resulting in a temporary or permanent suspension of a federally funded research project is reported to the grant program director responsible for the grant.
N/A

Describe plans for assuring data accuracy and protocol compliance.
To ensure accuracy of the collected data from study participants several procedures will be implemented. First, all study personnel will be involved in all testing to ensure measurement consistency across participants. All study personnel will be instructed by the PI on physical activity and continuous glucose monitoring technique. Questionnaires will be completed by study participants online via REDCap and will be checked by study personnel for clarification or missing information in the presence of the subject after completion. The study team will review the collected data on a weekly basis, to ensure that quality data are collected. We will utilize REDCap to manage our database. It allows us to construct user-friendly data collection forms with clearly labeled sections for each piece of data. In addition, REDCap flags any items that are beyond an acceptable range or that are inconsistent with acceptable values for categorical variables and it will check for missing values. This will reduce input error. However, these steps will not catch keypunch errors. Therefore, during data collection, we will have at least 2 research study staff check for potential errors and they will also immediately after each "completed "subject data collection will check for accuracy and missing information. REDCap access is password protected and resides on secure VU/VUMC server. By utilizing REDCap and having data reviewed on a frequent basis, we will be able to identify missing data quickly and attempt to collect it.

Is Vanderbilt going to be the Coordinating Center?
[ ] Yes
[**x**] **No**

Please select the phase of study.
[ ] Phase I
[ ] Phase I/II combined
[ ] Phase II
[ ] Phase III,
[ ] Phase IV
[**x**] **N/A**

Does this study require registration with clinicaltrials.gov?
[**x**] **Yes**
[ ] No

Subject Population(s)

Is this a study in which you will have interaction with individuals? NOTE: Please check "yes" if you need to report accrual goals for participant engagement.
[x] **Yes**
[ ] No

Accrual Goal: What is your total accrual goal at Vanderbilt?
70

Total number of participants stated in the protocol to be studied at all sites (regardless of PI).
70

Does this study target one gender or specific social/ethnic group(s)?
[ ] Yes
[x] **No**

Is the population being enrolled in this study at high risk for incarceration?
[ ] Yes
[x] **No**

Check all that apply (*Complete the appropriate supplemental information as applicable):
[x] **N/A**
[ ] Children/minors*
[ ] Cognitively impaired - comatose/traumatized*
[ ] Pregnant women/fetal tissue/placenta*
[ ] Prisoners*

Recruitment

Describe the specific steps to be used to identify and/or contact prospective participants. (If applicable, also describe how you have access to lists of potential participants.)
The subjects in this study will be recruited via online and offline. For online recruitment, we will use an email distribution list and ResearchMatch. In the email, there will be a link directing the potential participants to the prescreening e-consent via REDCap. They will have an option to download the e-consent for prescreening activities. After they sign on the prescreening e-consent, they will answer several questions about their eligibility (e.g. sitting hours > 8 hours a day). These pre-screening data will be saved into REDCap. Once they meet all of the inclusion criteria, the trained research staff will call them to confirm their eligibility and schedule an in-person baseline visit.

For offline recruitment, we will use two strategies. First, we will place our brochures and flyers in the VUMC Cardiology outpatient clinics. Second, our investigators (Dr. Jason and Dr. Hayes) who have an existing clinical relationship with their patients will distribute our brochures to their patients. If the potential participants are interested in our study, they will call the number on the recruitment flyers or brochures. For these calls, research personnel will be located in research offices. During the phone call, the trained research staff will answer any questions from the potential participants and provide them with the inclusion and exclusion criteria, allowing the participant to self-screen. If subjects are interested in participating and meet all of the eligibility criteria, an in-person baseline visit will be scheduled.

Identify the criteria for inclusion and exclusion and explain the procedures that will be used to determine eligibility. If psychiatric/psychological assessments will be conducted (e.g., depression or suicidal ideation screenings), state who will administer, his/her experience, and how risks will be managed.
Inclusion criteria: 1) ages 18 and above, 2) have at least one of the following conditions including history of heart attack or diagnosis of coronary artery disease or peripheral artery disease or a stent placed in heart or leg, 3) self-report of sitting ≥ 8hr/day, 4) ability to stand and walk, and 5) ownership of a smartphone.

Exclusion criteria: 1) currently using an activity tracker; 2) currently participating in exercise or cardiac rehabilitation programs; 3) non-English speaking; 4) patients who are classified as unstable (e.g. heart failure, uncontrolled arrhythmia) or have kidney disease that limits daily water intake, or any other conditions contradictory to standing or walking; and 5) currently pregnant.

Describe how the selection of participants is equitable in relation to the research purpose and setting.
Patients with cardiovascular disease remain highly sedentary and have prolonged patterns; they spend 10-14 hours per day in sedentary behavior, which comprises 70-90% of waking time and 50% of their total sedentary time is prolonged (>30 minutes per bout). Some (10-21%) cardiovascular disease patients engage in recommended levels of moderate-to-vigorous physical activity (MVPA), but their waking time is mostly occupied by SB. In our study, we select those who have a history of heart attack or diagnosed with coronary artery disease (= myocardial infarction), or diagnosed with peripheral artery disease (= peripheral vascular disease) and self-reported high sedentary (>8 hours of sitting per day) to test our Fitbit-based sedentary behavior reduction intervention program. For this study, no one ethnic group is targeted or excluded and the same group of participants will benefit from the results of the research.

Please indicate whether you plan to enroll any of the populations indicated below:
[ ] VU Medical Students/trainees
[ ] Students
[ ] Elderly/Aged - targeted
[ ] Subordinates/Employees
[ ] Females of childbearing potential
[ ] Terminally ill participants
[ ] Healthy Volunteers
[x] **Other**
[ ] Minorities

Please specify 'Other' populations:
Those who have at least one of the following conditions including 1) a history of heart attack, 2) diagnosed with coronary artery disease or peripheral artery disease, or 3) self-report of placing a stent or bypass in their heart or leg

Please identify ALL applicable recruitment methods:
[ ] N/A
[x] **Flyers**
[ ] Internet
[ ] Letter
[ ] Departmental Research Boards,
[x] **Mass E-mail Solicitation/Research Notifications Email Distribution List**
[ ] Newspaper
[x] **Posters**
[x] **ResearchMatch (IRB 090207)**
[ ] Radio
[ ] Telephone
[ ] Television
[ ] Social Media
[ ] EHR-Based Recruitment
[x] **EMR Recruitment - Existing provider relationship**
[ ] Other

Will the study provide compensation to research participants? Please be advised, any resident of a country subject to U.S. comprehensive territorial sanctions or any person designated on the U.S. Treasury Department's Office of Foreign Assets Control's Specially Designated Nationals list of prohibited individuals related to national security or foreign policy cannot accept any compensation for participation.
[x] **Yes**
[ ] No

Please specify the method of compensation.
Gift Certificates

Please include information describing the payment amount and schedule.
All participants will receive $50 on their first (Baseline visit 1), second visit (Baseline visit 2), and last visit (post-intervention visit) and exit-interview participants will receive an additional $30.
Intervention group participants will keep their Fitbit and Hidratespark smart water bottle (equivalent to $170).

Are you requesting a waiver for the collection of Social Security numbers?
[x] **Yes**
[ ] No

Does this study include a certificate of confidentiality or sensitive research information that must be hidden in the medical record?
[ ] Yes
[**x**] **No**

Radiation Procedures and Radioactive Drugs

Does this study involve any radiation ionizing procedures for research?
[ ] Yes
[**x**] **No**

Drugs, Devices, Biologics

Please check all that apply:
[x] **N/A**
[ ] Drug(s)/Biologic(s) or Placebo (inactive substance) Used for Research that HAVE an IND
[ ] Drug(s)/Biologic(s) or Placebo (inactive substance) used for Research that DO NOT have an IND
[ ] IBC Review for Live, Recombinant, and/or Attenuated Microorganisms for Vaccination, Gene Transfer or Botox
[x] **Device(s) Used for Research (devices may also include computer software, in vitro diagnostics, etc.)**

Please indicate each device in the study on a separate line in the table:

Name of Device	Manufacturer	Handling/Storage	FDA Determination	IDE Holder	IDE Exempt	IDE Number	Risk
Freestyle Libre Flash Glucose Monitoring System	Abbott, USA		510(K)				NSR
ActivPal3	PAL Technologies, UK		No FDA determination				NSR
Fitbit Inspire 2	Fitbit, USA		No FDA determination				NSR
HidrateSpark Steel Water Bottle	HidrateSpark, USA		No FDA determination				NSR

Please provide a description of each device:
]The FreeStyle Libre Pro Flash Glucose Monitoring System is an FDA-approved, professional continuous glucose monitoring device indicated for detecting trends and tracking patterns in persons (age 18 and older) with diabetes. After application of the sensor, only a thin, flexible filament will remain under the skin and the sensor is held in place by an adhesive. The sensor's data will be imported by a "reader" and the data will be saved to the PI's computer.

The activPAL activity tracker is a safe, non-invasive method to capture information about activity cycles. Participation in this study requires that participants wear an activPAL tracker taped (using Tegaderm adhesive dressing) to the front of the right thigh during waking hours for a minimum of 7 consecutive days (baseline and follow-up).

The Fitbit Inspire 2 is a small watch-type activity tracker, and data will be synced with the Fitbit app which is installed on the participants' smart phones.

The HidrateSpark water bottle is a stainless steel water bottle. The device alerts participants with a color change and message on the HidrateSpark app to keep participants hydrated. The device provides the daily water intake summary in the HidrateSpark app and also Fitbit app.

Please provide an explanation for each determination of non-significant risk.
The FreeStyle Libre Pro Flash Glucose Monitoring System is a FDA-approved, professional continuous glucose monitoring device. The following are possible non-significant risks of inserting a sensor and wearing the adhesive patch: local erythema (redness), local infection, inflammation, pain or discomfort, bleeding at the glucose sensor insertion site, bruising, itching, scarring or skin discoloration, and adhesive irritation. There is a remote risk of sensor or needle fracture during insertion, wear or removal, with fragments retained under the skin.

The activPAL activity tracker is a safe, non-invasive small device. There are few risks associated with wearing the activPAL device and include slight discomfort, such as light pressure from the activPAL or irritation from the waterproof dressing. However, there is no significant risk associated with the ActivPAL device use.

The Fitbit inspire 2 is a small watch type activity tracker. There are few non-significant risks associated with wearing the device and include slight discomfort, such as light pressure from the wristband or irritation from wearing a damp band after showering or swimming. There is no significant risk associated with the Fitbit device use.

The HidrateSpark water bottle is a stainless steel water bottle and there is no significant risk associated with using the water bottle.

Has any other IRB determined any of the device pose Significant Risk?
[ ] Yes
[**x**] **No**

Will a sham product and/or procedure be used in this study?
[ ] Yes
[**x**] **No**

Privacy

Will the VUMC IRB be acting as the Privacy Board for Research with respect to the review of Authorizations or Waiver of Authorizations?
[**x**] **Yes**
[ ] No

Are you requesting a Waiver of Authorization?
[ ] Yes
[**x**] **No**

Consent

Please indicate what you plan to do with regard to consent (check all that apply):
[x] **Consent**
[ ] Request Waiver or Alteration of Consent
[ ] Consent was obtained in another study

Please describe the specific steps for obtaining informed consent at your site and the procedures that will be utilized to protect the privacy of individuals.
Two e-informed consents will be used. First, an e-informed consent for pre-screening activity will be obtained via REDCap, from those who are recruited online. Those who are recruited from offline and directly call us from flyers or brochures will not sign the e-consent for prescreening activity, because the trained research staff will read the pre-screening questions and not record any of their responses. Second, the main e-informed consent will be obtained in-person and administrated by the principal investigator and/or the research staff through REDCap. Proper informed consent will be given according to previous training in responsible conduct of research, protection of human research participants, and good clinical practice. We will provide a physical copy of informed consent to the participants.

Does the person obtaining consent have an existing relationship with the participant(s)?
[ ] Yes
[x] **No**

Please describe any waiting periods between informing potential participants of the research and obtaining consent, if applicable.
Not applicable

Will surrogate consent be requested?
[ ] Yes
[x] **No**

How will non-English speaking participants be consented?
[ ] A translated written informed consent document in a language understandable to the participant. This should be an accurate translation of the full informed consent document (consider having a translator present during the consenting process should the participant have any questions).
[ ] Orally, using a qualified translator to translate the English informed consent document to the participant, and a translated short form in a language understandable to the participant. (See "Documentation of Informed Consent" at IRB Policy IV.B for details).
[x] **Enrolling only English speaking participants.**

Please provide a justification for only enrolling English speaking participants.
Since this is an early phase trial especially focusing on testing feasibility of the study, we will enroll only a limited number of subjects. Also we expect those who have limited English proficiency will rarely present to the Vanderbilt outpatient clinics considering the demographical distribution in Nashville or have registered in ResearchMatch or the email distribution list. Lastly, the majority of the study questionnaires are only available in English. Therefore, we will only enroll English-speaking participants.

Conflict of Interest Disclosure

Is there a potential conflict of interest for the Principal Investigator or key personnel? • The PI is responsible for assuring that no arrangement has been entered into where the value of the ownership interests will be affected by the outcome of the research and no arrangement has been entered into where the amount of compensation will be affected by the outcome of the research. • Assessment should include anyone listed as Principal Investigator, or other research personnel on page 1 of this application. Please note that ownership described below apply to the aggregate ownership of an individual investigator, his/her spouse, domestic partner and dependent children). Do not consider the combined ownership of all investigators.

[ ] Yes
[**x**] **No**
